# Complete genome sequences of *Geobacillus* sp. WCH70, a thermophilic strain isolated from wood compost

**DOI:** 10.1186/s40793-016-0153-y

**Published:** 2016-04-27

**Authors:** Phillip J. Brumm, Miriam L. Land, David A. Mead

**Affiliations:** C5-6 Technologies LLC, Fitchburg, Wisconsin USA; Oak Ridge National Laboratory, Oak Ridge, Tennessee USA; Lucigen Corporation, Middleton, Wisconsin USA; Great Lakes Bioenergy Research Center, University of Wisconsin, Madison, Wisconsin USA

**Keywords:** *Geobacillus* sp. WCH70, Wood compost, Thermophile, Transposons, Restriction-modification

## Abstract

*Geobacillus* sp. WCH70 was one of several thermophilic organisms isolated from hot composts in the Middleton, WI area. Comparison of 16 S rRNA sequences showed the strain may be a new species, and is most closely related to *G. galactosidasius* and *G. toebii*. The genome was sequenced, assembled, and annotated by the DOE Joint Genome Institute and deposited at the NCBI in December 2009 (CP001638). The genome of *Geobacillus* species WCH70 consists of one circular chromosome of 3,893,306 bp with an average G + C content of 43 %, and two circular plasmids of 33,899 and 10,287 bp with an average G + C content of 40 %. Among sequenced organisms, *Geobacillus* sp. WCH70 shares highest Average Nucleotide Identity (86 %) with *G. thermoglucosidasius* strains, as well as similar genome organization. *Geobacillus* sp. WCH70 appears to be a highly adaptable organism, with an exceptionally high 125 annotated transposons in the genome. The organism also possesses four predicted restriction-modification systems not found in other *Geobacillus* species.

## Introduction

Originally classified as members of the genus *Bacillus*, *Geobacillus* species were reclassified into a separate genus based on properties such as 16S rRNA gene sequence analysis, lipid and fatty acid analysis, phenotypic characterization, and DNA–DNA hybridization experiments [1]. *Geobacillus* species have been isolated from high-temperature oilfields [2], a corroded pipeline in an extremely deep well [3], American [4, 5] African [6] and Russian [7] hot springs, marine vents [8], and the Mariana Trench [9]. In addition to these extreme environments, *Geobacillus* species are commonly found in composting materials [10]. *Geobacillus*. sp. WSUCF1 [11], *G. galactosidasius* [12] and *G. toebii* [13] were isolated from high-temperature composts. The ability of *Geobacillus* species to thrive in these varied and often hostile environments suggests that these species possess enzymes suitable for applications in challenging industrial environments [14, 15]. As part of a program to identify organisms, we isolated *Geobacillus* species from a variety of composts in Middleton, WI. We report here the isolation and genome sequence of *Geobacillus* sp. WCH70, isolated from high-temperature wood compost.

## Organism information

### Classification and features

**Fig. 1:**
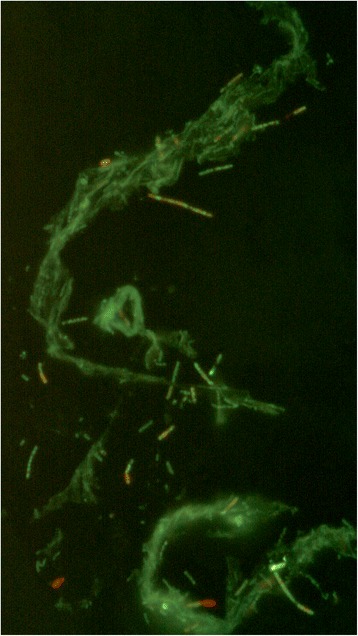
Micrograph of *Geobacillus* sp. Y412MC52 cells showing individual cells and clumps of cells. Cells were grown in TSB plus 0.4 % glucose for 18 h. at 70 °C. A 1.0 ml aliquot was removed, centrifuged, re-suspended in 0.2 ml of sterile water, and stained using a 50 μM solution of SYTO® 9 fluorescent stain in sterile water (Molecular Probes). Dark field fluorescence microscopy was performed using a Nikon Eclipse TE2000-S epifluorescence microscope at 2000× magnification using a high-pressure Hg light source and a 500 nm emission filter

*Geobacillus* sp*.*WCH70 is a novel thermophilic species isolated from a hot wood compost pile (~70 °C) in Middleton, WI (43.097090° latitude and -89.504730° longitude). The organism was isolated from a piece of decaying wood by enrichment and plating on YTP-2 medium (YTP-2 media contains (per liter) 2.0 g yeast extract, 2.0 g tryptone, 2.0 g sodium pyruvate, 1.0 g KCl, 2.0 g KNO_3_, 2.0 g Na_2_HPO_4_.7H_2_O, 0.1 g MgSO_4_, 0.03 g CaCl_2_, and 2.0 ml clarified tomato juice) at 70 °C. The culture is available from the *Bacillus* Genetic Stock Center. Cultures are routinely grown on tryptic soy broth without glucose (Difco) media and maintained on TSB agar plates. C5-6 Technologies, Lucigen, and the Joint Genome Institute have placed no restrictions on the use of the culture or sequence data. *Geobacillus* sp*.*WCH70 is a Gram-positive, rod-shaped facultative anaerobe (Table 1), with optimum growth temperature of 70 °C and maximum growth temperature of 80 °C. *Geobacillus* sp*.*WCH70 appears to grow as a mixture of single cells and large clumps in liquid culture (Fig. 1).

**Table 1:** Classification and general features of *Geobacillus* strain WCH70 [33]

MIGS ID	Property	Term	Evidence code^a^
	Classification	Domain *Bacteria*	TAS [34]
		Phylum *Firmicutes*	TAS [35]
		Class *Bacilli*	TAS [36, 37]
		Order *Bacillales*	TAS [38, 39]
		Family *Bacillaceae*	TAS [39, 40]
		Genus *Geobacillus*	TAS [1]
		Species *Geobacillus* sp.	
		Strain: WCH70	
	Gram stain	Positive	IDA
	Cell shape	Rods and chains of rods	IDA
	Motility	Motile	IDA
	Sporulation	Subterminal spores	IDA
	Temperature range	55 °C to 80 °C	IDA
	Optimum temperature	70 °C	IDA
	pH range; Optimum	5.8-8.0; 7.5	IDA
	Carbon source	Carbohydrate or protein	IDA
MIGS-6	Habitat	Compost	IDA
MIGS-6.3	Salinity	Not reported	IDA
MIGS-22	Oxygen requirement	Facultative anaerobe	IDA
MIGS-15	Biotic relationship	Free-living	IDA
MIGS-14	Pathogenicity	Non-pathogen	IDA
MIGS-4	Geographic location	Middleton, WI, USA	IDA
MIGS-5	Sample collection	September 2003	IDA
MIGS-4.1	Latitude	43.097090	IDA
MIGS-4.2	Longitude	-89.504730	IDA
MIGS-4.4	Altitude	342	TAS

^a^Evidence codes - IDA: Inferred from Direct Assay; TAS: Traceable Author Statement (i.e., a direct report exists in the literature); NAS: Non-traceable Author Statement (i.e., not directly observed for the living, isolated sample, but based on a generally accepted property for the species, or anecdotal evidence). These evidence codes are from the Gene Ontology project [41]

**Fig. 2:**
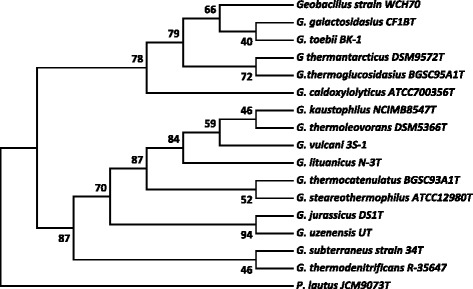
The evolutionary history was inferred by using the Maximum Likelihood method based on the Tamura-Nei model [18]. The bootstrap consensus tree inferred from 500 replicates [42] is taken to represent the evolutionary history of the taxa analyzed [42]. Branches corresponding to partitions reproduced in less than 50 % bootstrap replicates are collapsed. The percentage of replicate trees in which the associated taxa clustered together in the bootstrap test (500 replicates) are shown next to the branches [42]. Initial tree(s) for the heuristic search were obtained automatically by applying Neighbor-Join and BioNJ algorithms to a matrix of pairwise distances estimated using the Maximum Composite Likelihood (MCL) approach, and then selecting the topology with superior log likelihood value. The analysis involved 26 nucleotide sequences. All positions containing gaps and missing data were eliminated. There were a total of 1271 positions in the final dataset. Evolutionary analyses were conducted in MEGA5 [17]. The type strains of all validly described species are included (NCBI accession numbers): *G. caldoxylosilyticus* ATCC700356^T^ (AF067651), *G. galactosidasius* CF1B^T^ (AM408559), *G. jurassicus* DS1^T^ (FN428697), *G. kaustophilus* NCIMB8547^T^ (X60618), *G. lituanicus* N-3^T^ (AY044055), *G. stearothermophilus* R-35646^T^ (FN428694), *G. subterraneus* 34^T^ (AF276306), *G. thermantarcticus* DSM9572^T^ (FR749957), *G. thermocatenulatus* BGSC93A1^T^ (AY608935), *G. thermodenitrificans* R-35647^T^ (FN538993), *G. thermoglucosidasius* BGSC95A1^T^ (FN428685), *G. thermoleovorans* DSM5366^T^ (Z26923), *G. toebii* BK-1^T^ (FN428690), *G. uzenensis* U^T^ (AF276304) and *G. vulcani* 3S-1^T^ (AJ293805). The 16S rRNA sequence of *Paenibacillus lautus*JCM9073^T^ (AB073188) was used to root the tree

A phylogenetic tree was constructed to identify the relationship of *Geobacillus* sp*.*WCH70 to other members of the *Geobacillus* family (Fig. 2). The phylogeny of *Geobacillus* sp*.*WCH70 was determined using one of the ten16S rRNA gene sequence (genome coordinates 10256 through 11801), as well as those of the type strains of all validly described *Geobacillus* spp. The 16S rRNA gene sequences were aligned using MUSCLE [16], pairwise distances were estimated using the Maximum Composite Likelihood (MCL) approach, and initial trees for heuristic search were obtained automatically by applying the Neighbour-Joining method in MEGA 5 [17]. The alignment and heuristic trees were then used to infer the phylogeny using the Maximum Likelihood method based on Tamura-Nei [18]. Comparison of 16 S rRNA sequences shows *Geobacillus* sp*.*WCH70 clades with other 42 to 45 % G + C content species including *G. thermoglucosidasius*, *G. caldoxylolyticus,**G. galactosidasius* and *G. toebii* and is most closely related to *G. galactosidasius* and *G. toebii**.* Bootstrap analysis indicates that *G. galactosidasius* and *G. toebii* are more closely related to each other than to *Geobacillus* sp*.*WCH70, suggesting *Geobacillus* sp*.*WCH70 may be a new *Geobacillus* sp. Essentially identical trees were obtained when the other nine *Geobacillus* sp*.*WCH70 16S rRNA gene sequences were used to generate phylogenetic trees.

## Genome sequencing information

### Genome project history

*Geobacillus* sp*.*WCH70 was selected for sequencing on the basis of its biotechnological potential as part of the U.S. Department of Energy’s Genomic Science program (formerly Genomics:GTL). The genome sequence is deposited in the Genomes On Line Database [19, 20] (GOLD ID = Ga0028898), and in GenBank (NCBI Reference Sequence = CP001638.1). Sequencing, finishing and annotation were performed by the DOE JGI. A summary of the project information and its association with MIGS identifiers is shown in Table 2.

**Table 2:** Project information

MIGS ID	Property	Term
MIGS 31	Finishing quality	Finished
MIGS-28	Libraries used	8 Kb and 40 Kb
MIGS 29	Sequencing platforms	Sanger and 454
MIGS 31.2	Fold coverage	13 ×
MIGS 30	Assemblers	Phred/Phrap/Consed
MIGS 32	Gene calling method	Prodigal, GenePRIMP
	Locus Tag	GWCH70
	Genbank ID	NC_012793
	GenBank Date of Release	December 1, 2009
	GOLD ID	Gs0012167
	BIOPROJECT	PRJNA20805
MIGS 13	Source Material Identifier	Genome
	Project relevance	Biotechnological

### Growth conditions and genomic DNA preparation

For preparation of genomic DNA, one liter cultures of *Geobacillus* sp*.*WCH70 were grown from a single colony in YTP-2 medium at 70 °C in flasks agitated at 200 rpm and collected by centrifugation. Culture stocks were maintained on YTP-2 agar plates grown at 70 °C. The cell concentrate was lysed using a combination of SDS and proteinase K, and genomic DNA was isolated using a phenol/chloroform extraction. The genomic DNA was precipitated, and treated with RNase to remove residual contaminating RNA. The purity and concentration of the recovered DNA was determined by gel electrophoresis in 0.7 % agarose containing ethidium bromide. Low and high molecular weight lambda DNA ladders were used as standards. The purity,and quantity of the recovered DNA was also independently confirmed by the JGI as suitable for sequencing prior to initiation of the project.

### Genome sequencing and assembly

The genome of *Geobacillus* sp*.*WCH70 was sequenced at the JGI using a combination of Sanger and 454 technologies [21]. Two Sanger libraries with average insert size of 8 Kb and 40 Kb (fosmid) were generated for this genome. In addition to Sanger sequencing, 454 pyrosequencing was done to a depth of 20x coverage. Draft assemblies were based on 52,102 total reads. All three libraries provided 12.7x coverage of the genome. The Phred/Phrap/Consed software package was used for sequence assembly and quality assessment [22–24] in the following finishing process. After the shotgun stage, reads were assembled with parallel phrap (High Performance Software, LLC). Possible mis-assemblies were corrected with gapResolutioin (Cliff Han, unpublished), Dupfinisher, or sequencing cloned bridging PCR fragments with subcloning. Gaps between contigs were closed by editing in Consed, by PCR and by Bubble PCR primer walks. A total of 2,285 additional reactions were necessary to close gaps and to raise the quality of the finished sequence. The completed genome sequences of *Geobacillus* contains 56,142 reads, achieving an average of 13-fold sequence coverage per base with an error rate less than 1 in 100,000.

### Genome annotation

Genes were identified using Prodigal [25] as part of the Oak Ridge National Laboratory genome annotation pipeline, followed by a round of manual curation using the JGI GenePRIMP pipeline [26]. The predicted CDSs were translated and used to search the National Center for Biotechnology Information nonredundant database, UniProt, TIGRFam, Pfam, PRIAM, KEGG, COG, and InterPro databases. These data sources were combined to assert a product description for each predicted protein. Non-coding genes and miscellaneous features were predicted using tRNAscan-SE [26], RNAMMer [27], Rfam [28], TMHMM [29], and signalP [29].

## Genome properties

**Fig. 3:**
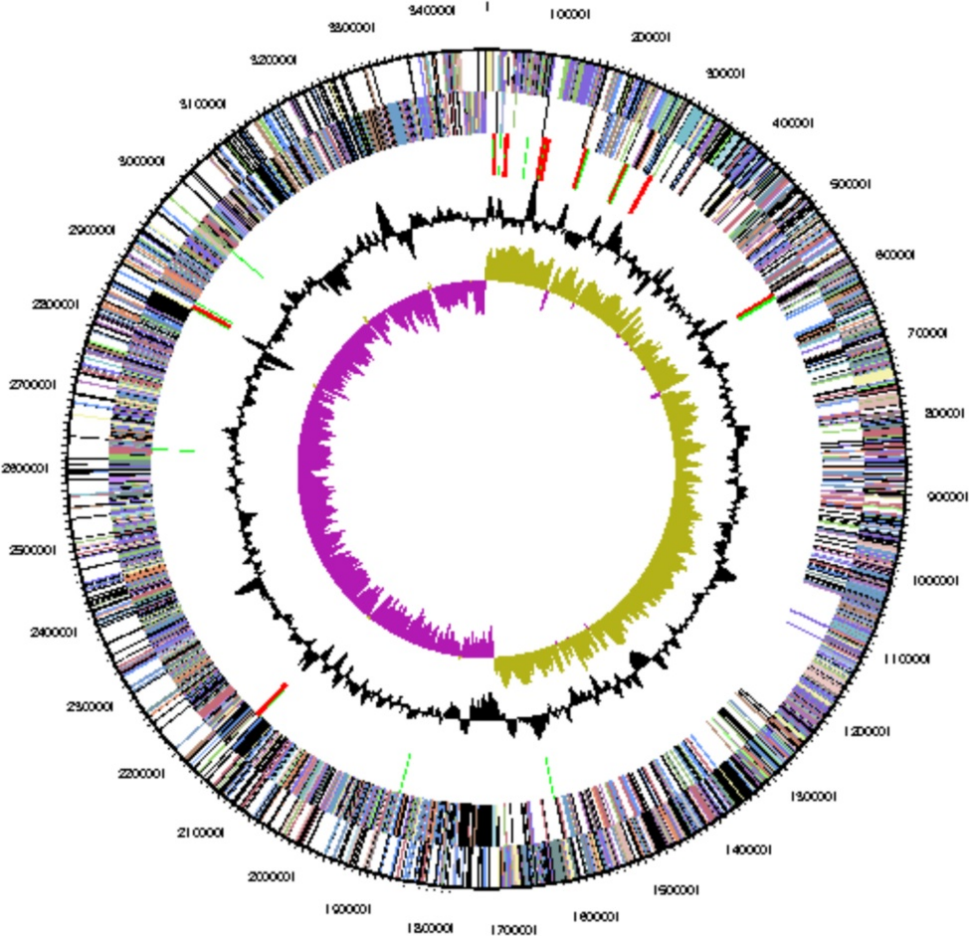
Graphical circular map of the *Geobacillus* sp*.* WCH70 chromosome. From outside to the center: Genes on forward strand (color by COG categories) Genes on reverse strand (color by COG categories) RNA genes (tRNAs green, rRNAs red, other RNAs black) GC content, GC skew

The genome of *Geobacillus* sp*.*WCH70 consists of one circular chromosome (Table 3 and Fig. 3) of 3,464,618 bp and an average G + C content of 43 % and two circular plasmids of 33,899 and 10,287 bp and an average G + C content of 40 % (Table 4). There are 92 tRNA genes and 28 rRNA genes. There are 3,477 predicted protein-coding regions and 309 pseudogenes in the genome. A total of 2,373 genes (66.0 %) have been assigned a predicted function while the rest have been designated as hypothetical proteins (Table 4). The numbers of genes assigned to each COG functional category are listed in Table 5. About 39 % of the annotated genes were not assigned to a COG or have an unknown function.

**Table 3:** Summary of genome: one chromosome and 2 plasmids

Label	Size (Mb)	Topology	INSDC identifier	RefSeq ID
Chromosome	3.46	Circular	CP001638.1	NC_012793
Plasmid 1	0.034	Circular	CP001639.1	NC_012794
Plasmid 2	0.010	Circular	CP001640.1	NC_012790

**Table 4:** Genome statistics

Attribute	Value	% of Total
Genome size (bp)	3,508,804	100.0
DNA coding (bp)	3,033,424	86.4
DNA G + C (bp)	1,501,708	42.8
DNA scaffolds	3	
Total genes	3597	100.0
Protein coding genes	3477	96.7
RNA genes	120	3.3
Pseudo genes	309	8.6
Genes in internal clusters		
Genes with function prediction	2373	66.0
Genes assigned to COGs	2201	61.2
Genes with Pfam domains	2946	81.9
Genes with signal peptides	125	3.5
Genes with transmembrane helices	805	22.4
CRISPR repeats	6

**Table 5:** Number of genes associated with general COG functional categories

Code	Value	%age	Description
J	195	8.0	Translation, ribosomal structure and biogenesis
A	0	0.0	RNA processing and modification
K	143	5.8	Transcription
L	94	3.8	Replication, recombination and repair
B	1	0.1	Chromatin structure and dynamics
D	102	4.2	Cell cycle control, Cell division, chromosome partitioning
V	65	2.6	Defense mechanisms
T	104	4.2	Signal transduction mechanisms
M	102	4.2	Cell wall/membrane biogenesis
N	62	2.5	Cell motility
U	33	1.4	Intracellular trafficking and secretion
O	97	4.0	Posttranslational modification, protein turnover, chaperones
C	140	5.7	Energy production and conversion
G	128	5.2	Carbohydrate transport and metabolism
E	222	9.1	Amino acid transport and metabolism
F	71	2.9	Nucleotide transport and metabolism
H	158	6.5	Coenzyme transport and metabolism
I	99	4.0	Lipid transport and metabolism
P	131	5.3	Inorganic ion transport and metabolism
Q	45	1.8	Secondary metabolites biosynthesis, transport and catabolism
R	194	7.9	General function prediction only
S	157	6.4	Function unknown
-	1396	38.8	Not in COGs

The total is based on the total number of protein coding genes in the genome

## Insights from the genome sequence

**Fig. 4:**
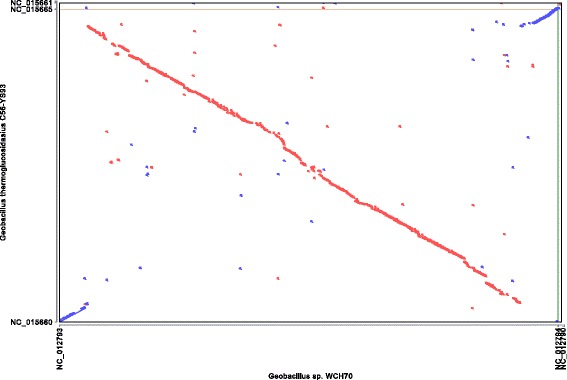
Synteny plot of *Geobacillus* sp*.* WCH70 versus *G. thermoglucosidasius* C56-YS93

The genome sequence of *Geobacillus* sp*.*WCH70 was compared to *Geobacillus* species with sequenced genomes. The lack of genome sequence information for *G. galactosidasius* and *G. toebii* prevents direct comparisons with these two organisms that are most closely related to *Geobacillus* sp*.*WCH70 based on rRNA gene sequences. *Geobacillus* sp*.*WCH70 Average Nucleotide Identity values [30] were 86.5 to 86.7 % to five *G. thermoglucosidasius* strains, 85.2 % to *G. stearothermophilus* NUB3621, and 84.9 and 85.0 % for two *G. caldoxylosilyticus* strains. ANI values ranged from 75.3 to 76.3 % for 20 other *Geobacillus* strains including *G. stearothermophilus*ATCC 7953, *G. thermodenitrificans*DSM 465, *G. subterraneus* PSS2, and *G. kaustophilus* HTA426. These values mirror the relationships of *Geobacillus* sp*.*WCH70 to other species seen in the phylogenetic tree based on rRNA. In addition to being closely related to *G. thermoglucosidasius* strains based on these two criteria, synteny plots reveal highly similar genome organizations in *Geobacillus* sp*.*WCH70 and *G. thermoglucosidasius* C56-YS93 (Fig. 4).

*Geobacillus* sp*.*WCH70 possesses a number of unusual features when compared to other *Geobacillus* species. A major feature of *Geobacillus* sp*.*WCH70 is the presence of 125 insertion-sequence (IS) elements predicted to code for transposons, significantly more elements than are found in sequenced strains of either *G. thermoglucosidasius* or *G. caldoxylosilyticus* (Table 6)*.* In addition to these IS elements, *Geobacillus* sp*.*WCH70 possesses four predicted restriction-modification gene clusters not found in other *Geobacillus* species. Genes GWCH70_1298 through GWCH70_1302 code for a predicted Type I restriction system most closely related to a system in *B. cereus* VD021, while GWCH70_2032 through GWCH70_2034 and GWCH70_3440 through GWCH70_3444 code for predicted Type I restriction systems most closely related to systems in *B. coagulans* XZL4. Genes GWCH70_2067 through GWCH70_2069 code for a predicted Type III restriction system most closely related to a system in *Thermincola ferriacetica*DSM 14005™*.* Genes GWCH70_1385 and GWCH70_1386 code for restriction system proteins most closely related to proteins in *Streptosporangium roseum*DSM 43021™. These restriction systems may facilitate transfer of DNA to and from other organisms in the compost microbiome.

**Table 6:** Comparison of predicted transposons

Function Name	COG id	WCH70	CIC9^a^	NBRC^b^	YU^c^	YS93^d^	GT20^e^	M10EXG^f^
Transposase, IS605 family	COG0675	62	3	2	0	1	0	0
REP element-mobilizing transposase RayT	COG1943	8	0	1	0	0	0	0
Transposase	COG3316	3	0	0	0	0	0	0
Transposase, mutator type	COG3328	15	0	1	4	7	4	3
Transposase, IS66 family	COG3436	10	0	0	0	0	0	0
Transposase, IS204 family	COG3464	9	0	0	0	0	0	1
Transposase, IS116 family	COG3547	11	0	1	0	5	0	1
Transposase	COG5421	7	0	0	0	0	0	0
Transposase	Not in WCH70	0	4	10	13	13	13	19
Total		125	7	15	17	26	17	24

^*a*^ *Geobacillus caldoxylosilyticus* CIC9, ^*b*^ *Geobacillus caldoxylosilyticus* NBRC 107762, ^*c*^ *Geobacillus thermoglucosidans* YU, ^d^ *Geobacillus thermoglucosidasius* C56-YS93, ^e^ *Geobacillus thermoglucosidasius* GT20, ^f^ *Geobacillus thermoglucosidasius* M10EXG, *Geobacillus thermoglucosidasius* NBRC 107763

Surprisingly, the genome of *Geobacillus* sp*.*WCH70 is lacking many of the predicted polysaccharide degradation clusters seen in other *Geobacillus* species [5], including the metabolic cluster for degrading hemicellulose [31]. The organism may utilize starch and other *alpha*-glucans based on the presence of a eleven-gene cluster GWCH70_0695 through GWCH70_0704 that is predicted to code for two, three-gene ABC carbohydrate transport systems, three *alpha*-amylase catalytic regions, an *alpha*-glucosidase, and a LacI family transcriptional regulator.

## Conclusions

*Geobacillus* sp*.*WCH70 is a thermophilic gram-positive, spore-forming organism isolated from hot wood compost in the Middleton, WI area. Comparison of 16 S rRNA sequences showed the strain may be a new species, and is most closely related to *G. galactosidasius* and *G. toebii*. The genome of *Geobacillus* has an average G + C content of 43 %, similar to that reported for *G. toebii* (43.9 %) [13]. *G. galactosidasius* is reported to possess a 53.5 % average G + C content [12] significantly higher than the value for *Geobacillus* sp*.*WCH70. Six *G. thermoglucosidasius* strains have 43.8 to 44.0 % average G + C content based on genomic sequence [32], similar to the value obtained for *Geobacillus* sp*.*WCH70. These G + C content values are lower than the 53 to 54 % obtained using chemical analyses [1, 12]. Genomic sequencing of *G. galactosidasius* and *G. toebii* is necessary to clarify the relationships among *Geobacillus* sp*.*WCH70, *G. galactosidasius* and *G. toebii**,* and *G. thermoglucosidasius**.*

The presence of 125 insertion-sequence (IS) elements predicted to code for transposons along with multiple restriction-modification systems suggests *Geobacillus* sp*.*WCH70 possesses a highly mutable chromosome, able to add or delete non-essential genes and gene clusters depending on the environmental conditions. Genomic sequencing of other *Geobacillus* species may help clarify if this mutability is a common element in other organisms in composts, or unique to *Geobacillus* sp*.*WCH70.

## Abbreviations

IMG: integrate microbial genomes database
JGI: joint genome institute
